# The Deletion of Several Amino Acid Stretches of *Escherichia coli* Alpha-Hemolysin (HlyA) Suggests That the Channel-Forming Domain Contains Beta-Strands

**DOI:** 10.1371/journal.pone.0112248

**Published:** 2014-12-02

**Authors:** Roland Benz, Elke Maier, Susanne Bauer, Albrecht Ludwig

**Affiliations:** 1 School of Engineering and Science, Jacobs University Bremen, Bremen, Germany; 2 Lehrstuhl für Mikrobiologie, Theodor-Boveri-Institut für Biowissenschaften (Biozentrum), Universität Würzburg, Würzburg, Germany; 3 Institut für Medizinische Mikrobiologie und Krankenhaushygiene, Klinikum der Johann Wolfgang Goethe-Universität, Frankfurt am Main, Germany; University of Alberta, Canada

## Abstract

*Escherichia coli* α-hemolysin (HlyA) is a pore-forming protein of 110 kDa belonging to the family of RTX toxins. A hydrophobic region between the amino acid residues 238 and 410 in the N-terminal half of HlyA has previously been suggested to form hydrophobic and/or amphipathic α-helices and has been shown to be important for hemolytic activity and pore formation in biological and artificial membranes. The structure of the HlyA transmembrane channel is, however, largely unknown. For further investigation of the channel structure, we deleted in HlyA different stretches of amino acids that could form amphipathic β-strands according to secondary structure predictions (residues 71–110, 158–167, 180–203, and 264–286). These deletions resulted in HlyA mutants with strongly reduced hemolytic activity. Lipid bilayer measurements demonstrated that HlyA_Δ71–110_ and HlyA_Δ264–286_ formed channels with much smaller single-channel conductance than wildtype HlyA, whereas their channel-forming activity was virtually as high as that of the wildtype toxin. HlyA_Δ158–167_ and HlyA_Δ180–203_ were unable to form defined channels in lipid bilayers. Calculations based on the single-channel data indicated that the channels generated by HlyA_Δ71–110_ and HlyA_Δ264–286_ had a smaller size (diameter about 1.4 to 1.8 nm) than wildtype HlyA channels (diameter about 2.0 to 2.6 nm), suggesting that in these mutants part of the channel-forming domain was removed. Osmotic protection experiments with erythrocytes confirmed that HlyA, HlyA_Δ71–110_, and HlyA_Δ264–286_ form defined transmembrane pores and suggested channel diameters that largely agreed with those estimated from the single-channel data. Taken together, these results suggest that the channel-forming domain of HlyA might contain β-strands, possibly in addition to α-helical structures.

## Introduction

Alpha-hemolysin (HlyA) of *Escherichia coli* is a member of a large family of cytolytic pore-forming toxins (PFTs) produced by a variety of Gram-negative bacteria. These toxins share common structural properties and contain in particular a series of glycine-rich nonapeptide repeats with the consensus sequence G–G-X-G-(N/D)-D-X-(L/I/F)-X (where X is any amino acid) in the C-terminal half of the toxin protein; they are therefore called RTX (Repeats in ToXin) toxins [1], [2]. The primary structure of the HlyA protein (1,024 amino acid residues) has several different domains. The C-terminus (about 50–60 residues) is essential for the secretion of HlyA out of the *E. coli* cell by a type I export system comprising the inner membrane components HlyB (a transport ATPase) and HlyD (adaptor protein) and the minor outer membrane channel-tunnel protein TolC [3]–[7]. The post-translational activation of HlyA involves the covalent fatty acylation of two lysine residues at position 564 and 690 by the cytoplasmic acyl-transferase HlyC [8]–[11]. The repeat domain (amino acid residues 724 to 852, comprising 13 nonameric repeats) binds Ca^2+^ and is essential for recognition of the mammalian target cell but it is not essential for channel formation in lipid bilayer membranes [12], [13]. A pronounced hydrophobic region in the N-terminal half of HlyA (residues 238 to 410), which may form hydrophobic and/or amphipathic α-helices, has rather been shown to be crucial for pore formation [14]–[19].

*E. coli* HlyA efficiently lyses erythrocytes and shows strong cytotoxic and cytolytic activity against a variety of nucleated cells [1], [2], [20]. The binding of HlyA to target cells has recently been suggested to be not receptor-dependent [21], but the issue of the presence or absence of HlyA receptors on different cells is still controversial [22]–[25]. Interestingly, HlyA does not only kill and lyse cells but it also affects target cells at sublytic concentrations. It has been shown, for example, that HlyA induces Ca^2+^ oscillations in renal epithelial cells leading to the production of pro-inflammatory cytokines [26]. In addition, it has been reported that these calcium oscillations depend on the influx of extracellular Ca^2+^ through L-type calcium channels in the plasma membrane and on the release of Ca^2+^ from stores within the endoplasmatic reticulum [26]. However, a more recent study suggested that they are caused by pulses of Ca^2+^ influx through short-lived HlyA pores that are rapidly closed or removed from the plasma membrane [27].

Secondary structure predictions suggested that the hydrophobic domain (residues 238–410) of the otherwise largely hydrophilic *E. coli* HlyA protein might contain hydrophobic and amphipathic α-helical structures that could be part of the transmembrane channel [15], [16]. The importance of this region for membrane insertion and pore formation has also been shown experimentally [14]–[19]. The channel structure itself is, however, still a matter of debate. Our own lipid bilayer studies suggest that several non-conductive HlyA monomers are needed to form a conductive oligomer, which has at low transmembrane potentials two well-defined conductance states: a small prestate and a transient open state following the prestate [28]. Oligomer formation was also suggested for other RTX toxins, such as the adenylate cyclase toxin (CyaA) of *Bordetella pertussis* or HlyA of the *Proteus* group [29]–[32]. In addition, complementation studies using different HlyA mutants suggested that oligomerization of *E. coli* HlyA is also a prerequisite for hemolysis [33]. However, vesicle studies also suggested that HlyA may act by a single hit mechanism [34]. Studies with erythrocytes and artificial membranes have demonstrated that a pore with a diameter of 1–3 nm is formed by HlyA [20], [28], [35]. Nevertheless, investigations of channel formation by HlyA in red blood cells also have suggested that the hemolytic activity of HlyA may not be due to the generation of defined channels in the erythrocyte membrane but rather to a detergent-like action of the toxin [36]. This has been concluded from the experimental observation that in osmotic protection experiments larger solutes are needed for protection at higher hemolysin concentrations and at longer assay times. Similarly, a detergent-like effect of HlyA on lipid vesicles formed from lecithin has been suggested from spectroscopic studies [37]. Again, no indication for a defined or any transmembrane arrangement of the toxin has been found in these studies, which suggested that HlyA occupies only one of the two phospholipid monolayers of the membrane. A transmembrane organization of HlyA has also not been recognized in electron microscopic analyses [37].

In this study, we describe lipid bilayer measurements and osmotic protection experiments using HlyA mutants in which four stretches of amino acids within the first 300 residues (residues 71–110, 158–167, 180–203, and 264–286) were deleted. These stretches contain one or two putative amphipathic β-strands of at least about 9 amino acid residues according to secondary structure predictions ([38], programs PRED-TMBB (http://biophysics.biol.uoa.gr/PRED-TMBB/) and TMBETA-NET (http://psfs.cbrc.jp/tmbeta-net/)), and many of the residues within these stretches are highly conserved among different RTX toxins. All four deletions strongly impaired or almost abolished the hemolytic activity of HlyA, but two of the HlyA mutants (HlyA_Δ71–110_ and HlyA_Δ264–286_) were still able to form defined ion-permeable channels in lipid bilayers. Furthermore, the lipid bilayer data as well as the results of osmotic protection experiments with erythrocytes indicated that these channels are considerably smaller as compared to those formed by the wildtype toxin, suggesting that amphipathic β-strands may be involved in channel formation by HlyA. Control experiments with aerolysin from *Aeromonas sobria*, a cytolysin known to form defined channels [39], suggested that the osmotic protection depends on the concentration of the channel-forming components, even if they form a discrete-sized channel.

## Materials and Methods

### Bacterial strains, plasmids, and culture conditions

The *hlyCABD* operon required for synthesis and secretion of *E. coli* α-hemolysin was originally cloned from plasmid pHly152 [40]. pANN202–812 and pANN202–312* are recombinant derivatives of pBR322 and pACYC184, respectively, carrying this operon on a 16.7-kb *Sal*I insert [11], [41]. Plasmid pANN202–312 [40] is a pACYC184 derivative carrying the four *hly* genes on a 13.4-kb *Hin*dIII-*Sal*I insert that lacks part of the regulatory sequences present upstream from *hlyC*. All plasmids were propagated in *E. coli* 5K (Sm^r^ *lacY1 tonA21 thr-1 supE44 thi* r_k_^–^ m_k_^+^). The *E. coli* strains JM109, BMH71–18*mut*S and MK30–3 [12] were used as host strains for vectors of the M13mp series (New England Biolabs) that were employed for site-directed mutagenesis. All bacterial strains used in this study were grown aerobically at 37°C in double-concentrated yeast extract-tryptone (2×YT) medium (yeast extract [Difco], 10 g/liter; tryptone [Difco], 16 g/liter; NaCl, 10 g/liter) or on YT medium solidified with 1.5% (wt/vol) agar. Blood agar plates were prepared from YT medium supplemented with 4% defibrinated sheep blood (Oxoid). Antibiotics were used at the following final concentrations: ampicillin, 100 µg/ml; chloramphenicol (Cm), 30 µg/ml.

### Construction of HlyA_Δ71–110_, HlyA_Δ158–167_, HlyA_Δ180–203_, and HlyA_Δ264–286_

Deletion of the codons 71 to 110, 158 to 167, 180 to 203, and 264 to 286 of *hlyA*, coding for the amino acid stretches A**DELGIEVQY**DEKNG**TAITKQVFGTAEKLIGLTERGVTI**F, S**SMKIDELI**K, LAKA**SIELINQLVDTVA**SLNNNVN, and DTRTKAAA**GVELTTKVLGNV**GKG, respectively [42], [43], was performed by site-directed mutagenesis using the gapped duplex DNA approach [44]. (The putative amphipathic β-strands derived according to the programs PRED-TMBB [http://biophysics.biol.uoa.gr/PRED-TMBB/] and TMBETA-NET [http://psfs.cbrc.jp/tmbeta-net/] are given in bold and are underlined in the above sequences). A recombinant M13mp9 derivative with a 2.65-kb *Bam*HI-*Bgl*II insert spanning the 3′-terminal region of *hlyC* and the 830 5′-terminal codons of *hlyA* was used as DNA template for the generation of the deletions, and the mutagenesis was directed by the oligonucleotides shown in Table 1. Successful introduction of the deletions was verified by DNA sequencing (dideoxynucleotide chain termination method), using the T7 sequencing kit from Pharmacia. To transfer the deletions into the *hlyCABD* operon, a *Bam*HI-*Sph*I subfragment of the *Bam*HI-*Bgl*II insert containing the respective deletion was isolated and substituted for the corresponding 1.9-kb wildtype *Bam*HI-*Sph*I fragment in pANN202–312. To complete the *hlyCABD* operon, a 5.2-kb *Bam*HI fragment from pANN202-812, carrying the entire regulatory region of this operon and the 5′-terminal region of *hlyC*, was inserted into the unique *Bam*HI site present in *hlyC* of the mutant pANN202–312 derivatives, resulting in the plasmids pANN202–312*Mut37 (encoding HlyA_Δ71–110_), pANN202-312*Mut70 (HlyA_Δ158–167_), pANN202–312*Mut71 (HlyA_Δ180–203_), and pANN202-312*Mut72 (HlyA_Δ264–286_). The presence of the respective deletion in the *hlyA* gene of these plasmids was confirmed by DNA sequencing. DNA cloning procedures were generally carried out using standard protocols [45].

**Table 1:** Primers used for site-directed mutagenesis to study the effect of deletions of different stretches of amino acids of HlyA on hemolytic activity and properties of the HlyA channels.

HlyA mutant	Mutagenic oligonucleotide a
HlyA_Δ71–110_	5’-CTTGTCAGGACG|GCACCACAATTAG-3’
HlyA_Δ158–167_	5’-GGTACTGCACTT|AAACAAAAATCTG-3’
HlyA_Δ180–203_	5’-GTTCTTCTGAA|TCATTTTCTCAAC-3’
HlyA_Δ264–286_	5’-CAATGCAGATGCA|ATTTCTCAATAT-3’

a The 5′- and 3′-terminal halves of the primers represent the nucleotide sequences of *hlyA* flanking the desired deletions on both sides. The deletion site is indicated by a vertical bar (|).

### Isolation and purification of wildtype and mutant HlyA

**Figure 1:**
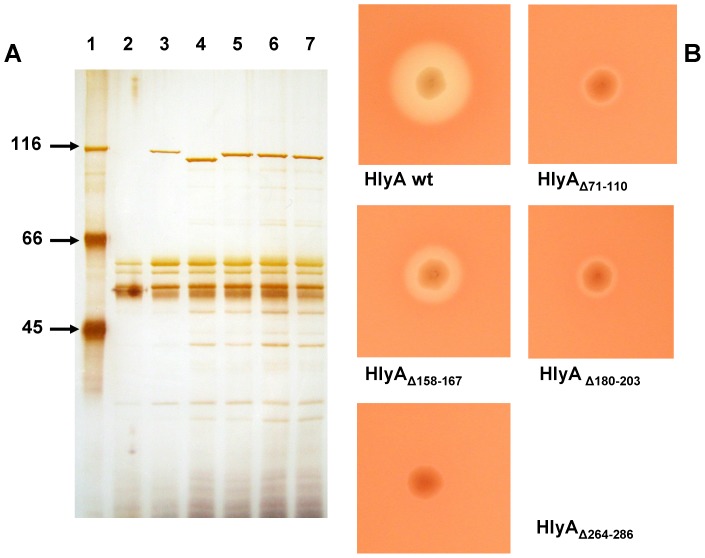
Extracellular secretion and hemolytic activity of *E. coli* HlyA and of HlyA mutants. (A) SDS-PAGE of extracellular proteins from *E. coli* 5K containing different plasmids. Lane 1, molecular mass markers given in kDa; lane 2, *E. coli* 5K/pACYC184 (vector control); lane 3, *E. coli* 5K/pANN202–312* overproducing HlyA; lane 4, isogenic strain overproducing HlyA_Δ71–110_; lane 5, isogenic strain overproducing HlyA_Δ158–167_; lane 6, isogenic strain overproducing HlyA_Δ180–203_; lane 7, isogenic strain overproducing HlyA_Δ264–286_. The proteins in cell-free culture supernatants (harvested in the late log phase) were precipitated by addition of ice-cold trichloroacetic acid (final concentration, 10%), pelleted by centrifugation at 12,000×g, washed with acetone, dried under vacuum, and dissolved in sample buffer [11]. Proteins from 100 µl culture supernatant were separated on the gel and visualized by silver staining. (B) Hemolytic phenotype of *E. coli* 5K/pANN202–312* overproducing HlyA and of isogenic strains overproducing the HlyA mutants with the indicated deletions. Bacteria from individual colonies were picked onto a sheep blood/Cm agar plate that was subsequently incubated for 24 hours at 37°C.

**Figure 2:**
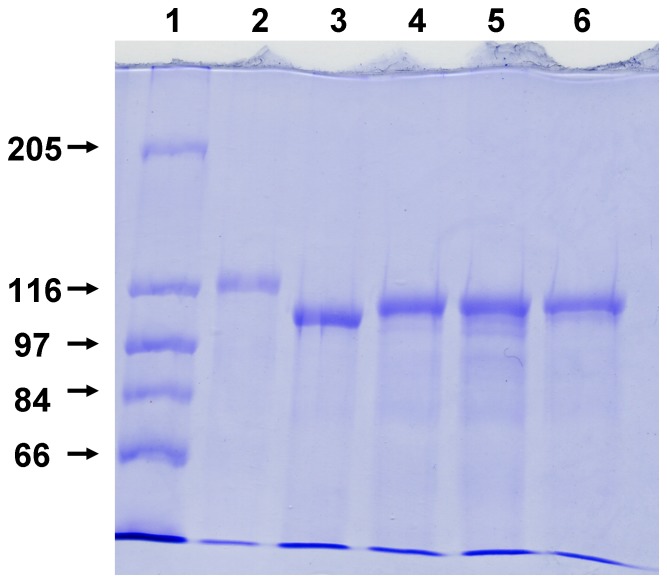
SDS-PAGE of purified *E. coli* HlyA and of HlyA mutants. Wildtype and mutant HlyA were expressed in *E. coli* 5K/pANN202–312* and isogenic mutant strains, respectively, and purified from culture supernatants using preparative SDS-PAGE. Lane 1, molecular mass markers given in kDa; lane 2, HlyA; lane 3, HlyA_Δ71–110_; lane 4, HlyA_Δ158–167_; lane 5, HlyA_Δ180–203_; lane 6, HlyA_Δ264–286_. In each lane, 5 µg of protein was separated and visualized by Coomassie blue staining.

Identical aliquots of fresh growth medium (20 ml) were inoculated 1∶100 with overnight cultures of *E. coli* 5K harboring either the wildtype plasmid pANN202–312* or the mutant plasmids pANN202–312*Mut37, -Mut70, -Mut71, and -Mut72. The cultures were grown at 37°C with agitation until an OD_550_ of 1.4 was reached. The cells were harvested by centrifugation for 15 min at 8,000 rpm in a pre-cooled Beckman JA-17 rotor. The cell-free supernatants containing either HlyA or the HlyA mutants were kept on ice and were used for hemolysis liquid assays or lipid bilayer experiments without further purification (Fig. 1A). Alternatively, proteins were precipitated with 18% polyethylen glycol (PEG) 4000 and redissolved in 10 mM Tris-HCl, pH 7.0. The final purification of wildtype and mutant HlyA was achieved by preparative sodium dodecyl sulfate-polyacrylamide gel electrophoresis (SDS-PAGE) [13]. The eluted proteins were kept in 8 M Urea, 50 mM Tris-HCl pH 8.0, 2 mM EGTA and stored at −20°C. It is noteworthy that HlyA and its mutants were stable under these conditions for at least three months. Purified HlyA and its mutants were essentially free of contaminant proteins as illustrated in Fig. 2.

### Purification of aerolysin

Aerolysin was isolated from supernatants of *Aeromonas sobria* AB3 cultures as has been described previously [39].

### Gel electrophoresis of proteins and immunoblot analysis

The concentration of HlyA and its mutants was measured as OD_280_. SDS-PAGE of proteins was performed as described by Laemmli [46]. For immunoblot analysis, proteins separated by SDS-PAGE were transferred to Hybond N membrane (Amersham) according to Towbin *et al.* [47]. The proteins were probed with a polyclonal rabbit anti-HlyA antiserum [48] that was used in a dilution of 1∶1,000. Proteins reacting with this antiserum were detected by addition of horseradish peroxidase-conjugated anti-rabbit immunoglobulins (Dianova, dilution 1∶1,000) followed by colorimetric development with chloronaphthol/H_2_O_2_.

### Hemolysis assay and osmotic protection experiments

The extracellular hemolytic activities of *E. coli* strains and the relative hemolytic activities of wildtype and mutant HlyA were determined as described previously [11]. For osmotic protection experiments, sheep erythrocyte suspensions (2%) were prepared in saline solution (150 mM NaCl, 5 mM Tris-HCl buffer (pH 7.2)) containing one of the following carbohydrates in a final concentration of 30 mM: arabinose (molecular mass 150.4 Da, diameter 0.62 nm; Sigma, St. Louis, MO), cellobiose (molecular mass 342.3 Da, diameter 0.92 nm; Sigma), melezitose (molecular mass 504.4 Da, diameter 1.14 nm; Sigma), as has been described previously [20], [36]. In additional measurements, we used the following PEGs in osmotic protection experiments: PEG 400 (diameter 1.07 nm), PEG 600 (diameter 1.32 nm), PEG 1000 (diameter 1.72 nm), PEG 2000 (diameter 2.47 nm), PEG 3000 (diameter 3.05 nm), and PEG 4000 (diameter 3.54 nm), all in the same concentration as the carbohydrates. For the protection experiments, the sheep erythrocytes were incubated for different times at 37°C with *E. coli* HlyA, HlyA mutants, or aerolysin. The lysis of the erythrocytes was quantified by hemoglobin release as determined at OD_543_. The results were expressed in percentage of total hemoglobin release.

### Black lipid membrane experiments

Reconstitution of channel-forming proteins into artificial lipid bilayer membranes has been described previously in detail [49]. Membranes were formed from a 1% (mass/vol) solution of asolectin (soybean lecithin type IV-S from Sigma, St. Louis, MO) in n-decane in a Teflon cell consisting of two aqueous compartments connected by a circular hole with a surface area of about 0.2 mm^2^. Small amounts of the concentrated hemolysin solutions were added to 5 ml of the aqueous phase at one or both sides of the membrane to yield hemolysin concentrations between 0.01 and 1 µg/ml. The aqueous salt solutions (analytical grade, Merck, Darmstadt, Germany) were used unbuffered and had a pH around 6 if not indicated otherwise. The temperature was kept at 20°C throughout. The membrane current was measured with a pair of Ag/AgCl electrodes switched in series with a voltage source and a current amplifier (Keithley 427). The amplified signal was recorded with a strip chart recorder. Zero-current membrane potential measurements were performed as described earlier [50].

### Estimation of the diameter of the channels formed by wildtype and mutant HlyA

The channel size can be calculated from the conductance data when the ions move through the channel similar as in the aqueous phase and when the entry of a hydrated ion into the effective area, *A*, of the channel mouth is the rate-limiting step (and not the diffusion of the hydrated ion through the channel itself). The estimation is based on the same assumptions that were used previously for the derivation of the Renkin correction factor [51] for the diffusion of neutral molecules through porous membranes and porin channels [51], [52], and for the diffusion of ions through wide and water-filled ion channels [53]. The permeability of a cylindrical channel (radius *r*) for solutes is proportional to their aqueous diffusion coefficient, *D*, multiplied with the Renkin correction factor given by:
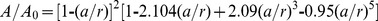
(1)where *A* is the effective area of the channel mouth, *A*_0_ is the total cross sectional area of the channel, and 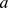 is the radius of the hydrated ions or substrates passing through the channel. To apply the Renkin correction factor for the calculation of the channel size, we have to know the radii of the hydrated ions, 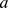, and their diffusion coefficients, *D*, in the aqueous phase, being also a function of the hydrated ion radii. The radii of the hydrated ions can be calculated from the limiting molar conductivities, *λ_i_*, of the ions by using the Stokes equation:
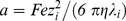
(2)where *F* (*F* = 96500 As/mol) is the Faraday constant, *e* (*e* = 1.602⋅10^–19^ A⋅s) is the elementary charge, *z_i_* is the valency of the ions, and *η* (*η* = 1.002⋅10^−3^ kg/(m⋅s)) is the viscosity of the aqueous phase. The validity of the method has previously been assessed by comparing the size of the cell wall channel of *Mycobacterium chelonae* as estimated from the method described above (i.e. from the single-channel conductance) and from the vesicle-swelling assay using OmpF of *E. coli* [52], [53].

## Results

### Secretion and hemolytic activity of the HlyA mutants HlyA_Δ71–110_, HlyA_Δ158–167_, HlyA_Δ180–203_, and HlyA_Δ264–286_

*E. coli* 5K clones containing the plasmids pANN202–312*Mut37, -Mut70, -Mut71, and -Mut72, which encode HlyA_Δ71–110_, HlyA_Δ158–167_, HlyA_Δ180–203_, and HlyA_Δ264–286_, respectively, specifically secreted proteins with molecular masses between about 105 and 110 kDa into the extracellular medium, as shown by SDS-PAGE of culture supernatants (Fig. 1A). The sizes of these proteins were consistent with the molecular masses calculated for the four HlyA mutants. In addition, these proteins reacted with a polyclonal anti-HlyA antiserum (not shown), indicating that they were indeed the HlyA mutants encoded by the plasmids. The concentration of these HlyA mutants in the culture supernatants of the recombinant *E. coli* 5K clones was approximately the same as that of wildtype HlyA (molecular mass, 110 kDa) found under identical conditions in the supernatant of *E. coli* 5K/pANN202–312* (about 5 µg/ml in the late log phase; see Fig. 1A) [11], [15]. This demonstrated efficient secretion of the different HlyA mutants by the HlyA export apparatus.

The *E. coli* 5K clones overproducing HlyA_Δ71–110_ and HlyA_Δ180–203_ exhibited only a very weak hemolytic phenotype when grown overnight on blood agar plates, while the isogenic wildtype strain *E. coli* 5K/pANN202–312* produced large, clear lysis zones under the same conditions (Fig. 1B). In hemolysis assays using culture supernatants from both strains, the relative hemolytic activity of HlyA_Δ71–110_ and HlyA_Δ180–203_ was about 1% as compared to that of wildtype HlyA. HlyA_Δ158–167_ had a somewhat higher hemolytic activity than HlyA_Δ71–110_ but it was definitely much smaller than that of wildtype HlyA. The hemolytic activity of the fourth HlyA mutant, HlyA_Δ264–286_, was extremely low. Only on blood agar plates stored for some days at 4°C after growth of the bacterial colonies, we observed some hemolysis beneath the colonies (Fig. 1B). It is noteworthy that the hemolytic activity of HlyA_Δ71–110_, HlyA_Δ158–167_, and HlyA_Δ180–203_ relative to wildtype HlyA was the same irrespective if culture supernatants, protein concentrated by PEG precipitation or purified protein (Fig. 2) was used in the experiments provided the toxin concentration in the aqueous phase was the same.

### HlyA_Δ71–110_ and HlyA_Δ264–286_ form ion-permeable channels in lipid bilayer membranes

**Figure 3:**
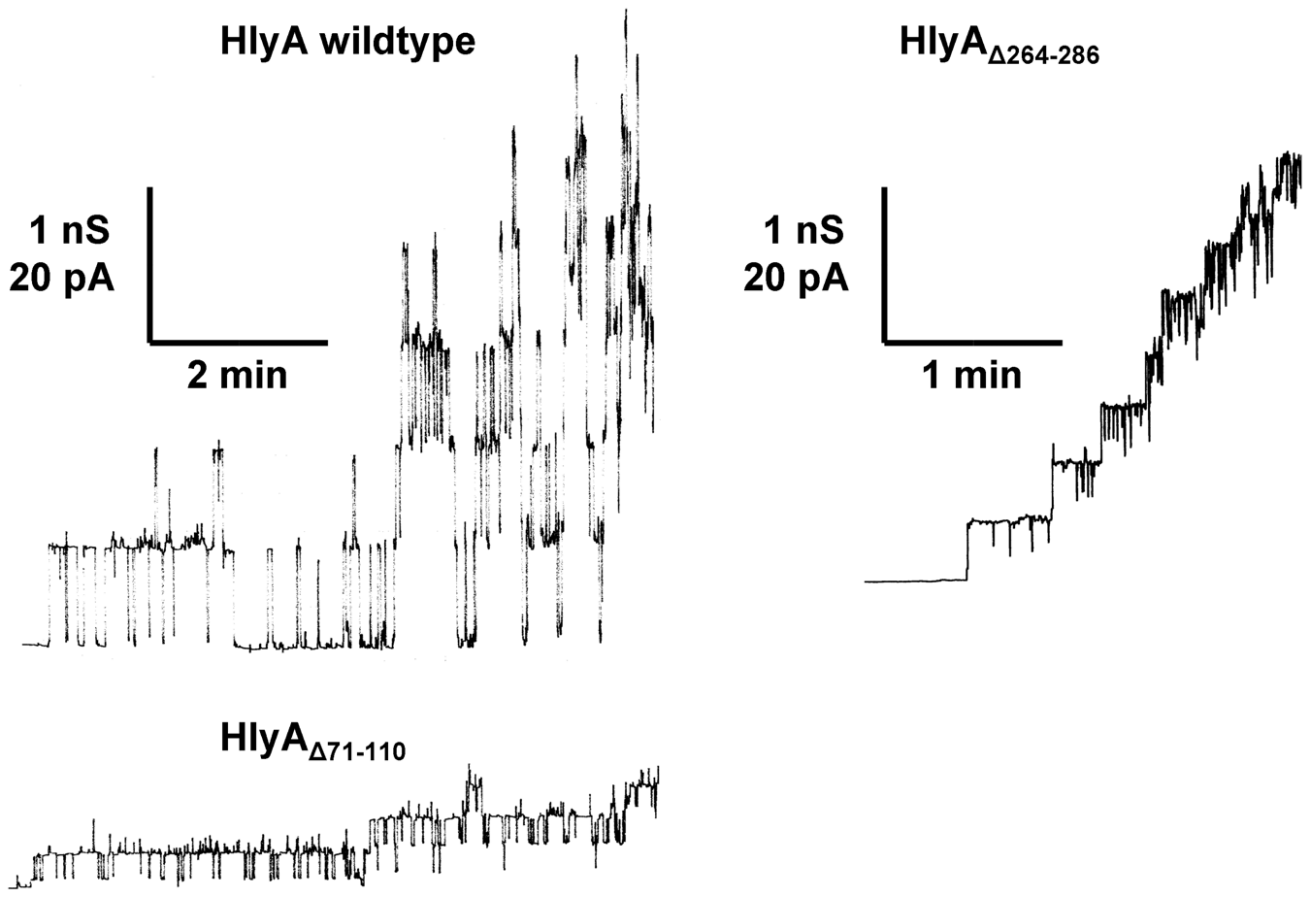
Single-channel recordings with *E. coli* HlyA, HlyA_Δ71–110_, and HlyA_Δ264–286_. Single-channel recordings of asolectin membranes were performed in the presence of 50 ng/ml HlyA (left side, upper trace), 50 ng/ml HlyA_Δ71–110_ (left side, lower trace), and 50 ng/ml HlyA_Δ264–286_ (right side). The aqueous phase contained 150 mM KCl (pH 6). The applied membrane potential was 20 mV; T = 20°C. The average single-channel conductance was 520 pS for HlyA, 150 pS for HlyA_Δ71–110_, and 320 pS for HlyA_Δ264–286_.

We performed single-channel experiments with all four HlyA mutants. The experiments revealed that HlyA_Δ71–110_ and HlyA_Δ264–286_ still formed defined channels in lipid bilayer membranes but with much lower amplitude (i.e. single-channel conductance) than wildtype HlyA under otherwise identical conditions. Fig. 3 shows single-channel recordings of asolectin/n-decane membranes in the presence of wildtype HlyA, HlyA_Δ71–110_, and HlyA_Δ264–286_ in 150 mM KCl. All proteins were added to black membranes in a concentration of about 50 ng/ml. After a delay of several minutes, probably caused by slow aqueous diffusion and/or rearrangement of the toxin, we observed for HlyA and the two mutants the occurrence of transient ion-permeable channels. This means that the channels formed by HlyA_Δ71–110_ and HlyA_Δ264–286_ also had a limited lifetime (mean lifetime about 4 s) similar to wildtype HlyA [28]. Wildtype HlyA channels had a single-channel conductance, *G*, of about 520 pS, whereas the channels formed by HlyA_Δ71–110_ and HlyA_Δ264–286_ had with about 150 pS and 320 pS, respectively, a much smaller one (all in 150 mM KCl and at 20 mV membrane potential). The channel-forming activity was approximately the same for HlyA, HlyA_Δ71–110_, and HlyA_Δ264–286_, which means that at the same toxin concentration approximately the same number of channels was observed irrespective whether culture supernatants, precipitated proteins or pure toxins were added to the aqueous phase. The results thus indicated that these two mutations had only little influence on channel-forming probability or channel lifetime and affected only the single-channel conductance (i.e. the size) of the HlyA channel.

### HlyA_Δ158–167_ and HlyA_Δ180–203_ showed some membrane activity but do not form defined ion-permeable channels in lipid bilayer membranes

**Figure 4:**
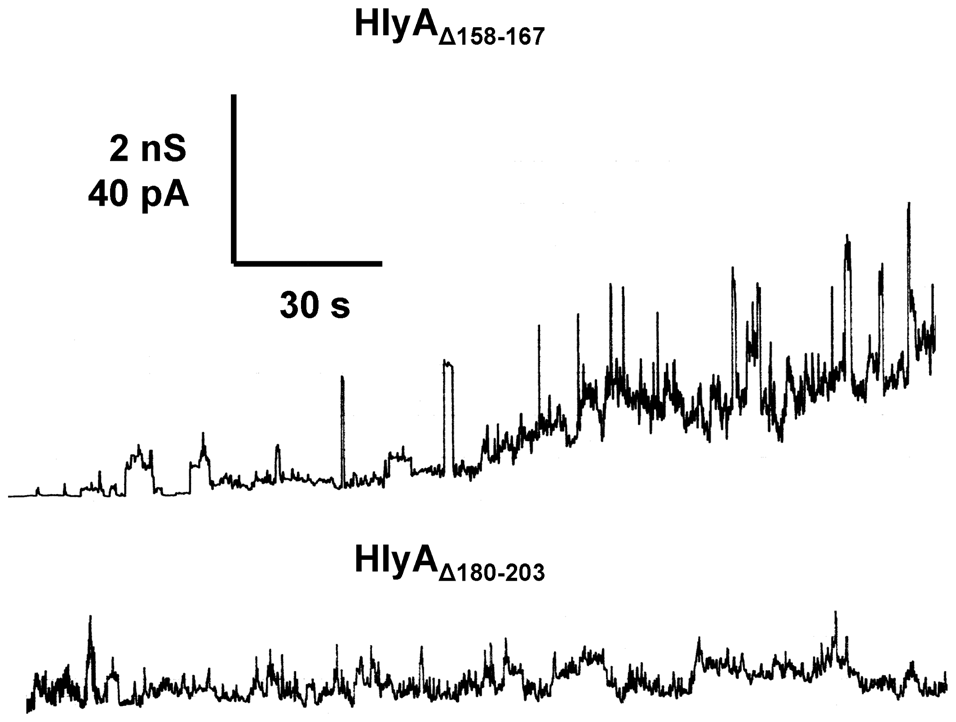
Single-channel recordings with HlyA_Δ158–167_ and HlyA_Δ180–203_. Single-channel recordings of asolectin membranes were performed in the presence of 100 ng/ml HlyA_Δ158–167_ (upper trace) and 150 ng/ml HlyA_Δ180–203_ (lower trace). The aqueous phase contained 150 mM KCl (pH 6). The applied membrane potential was 20 mV; T = 20°C. The transient conductance steps in the upper trace (HlyA_Δ158–167_) had a conductance of about 600 to 700 pS. The mutant HlyA_Δ180–203_ produced under the given conditions only current noise (fuzzy channels) and no defined conductance states.

Single-channel experiments were also performed with the other two HlyA deletion mutants. However, for these mutants we did not observe defined channels similar to those formed by HlyA, HlyA_Δ71–110_, or HlyA_Δ264–286_. Instead, HlyA_Δ158–167_ created current noise with flickers and bursts in the asolectin/n-decane membranes interrupted by transient and short-lived channels with amplitudes around 600 to 700 pS in 150 mM KCl (Fig. 4, upper trace). The membrane activity of this mutant was moderate, which means that a higher concentration of HlyA_Δ158–167_ was needed to observe effects at the asolection membranes. HlyA_Δ180–203_ had an even lower effect on membrane conductance. Only some membrane activity in the form of current noise but no defined membrane channels were observed at very high concentration of HlyA_Δ180–203_ (Fig. 4, lower trace).

### Single-channel conductance of the HlyA_Δ71–110_ and HlyA_Δ264–286_ channels

We performed also single-channel experiments with a variety of salts and concentrations to obtain some information on the size and the ion selectivity of the channels formed by HlyA_Δ71–110_ and HlyA_Δ264–286_. Table 2 shows the results of these experiments together with the single-channel data that have been derived previously from similar experiments with wildtype HlyA [28] or that were measured in this study using purified HlyA. The replacement of chloride (Cl^-^) by the less mobile acetate (CH_3_COO^-^) had only a little if any influence on the single-channel conductances of HlyA, HlyA_Δ71–110_, and HlyA_Δ264–286_, indicating that the mutant hemolysin channels were still highly cation-selective. The influence of the cations on the single-channel conductance of both mutants was more substantial. The ion selectivity of the HlyA_Δ71–110_ channel was Cs^+^ = Rb^+^ = K^+^> Na^+^> Li^+^> N(CH_3_)_4_^+^> N(C_2_H_5_)_4_^+^ = Tris^+^, which means that it followed the mobility sequence of the ions in the aqueous phase. Table 2 also shows the average single-channel conductance, *G*, as a function of the KCl concentration in the aqueous phase for HlyA, HlyA_Δ71–110_, and HlyA_Δ264–286_. Surprisingly, we neither observed for HlyA, nor for the two HlyA mutants a linear relationship between conductance and KCl concentration, which would be expected for wide, water-filled channels similar to those formed by general diffusion pores of Gram-negative bacteria [54]. Instead, the slope of the conductance versus concentration curves on a double logarithmic scale was approximately 0.5, which suggested negative surface charge effects on the hemolysin channels (see Discussion).

**Table 2:** Average single-channel conductance, *G*, of HlyA_Δ71–110_ and HlyA_Δ264–286_ in different salt solutions.^a.^

			*G*[nS]	
Salt	*c*[M]	HlyA_Δ71–110_	HlyA_Δ264–286_	HlyA
LiCl	0.15	0.045	0.074	0.15
	1.0	0.13	n.m.	n.m.
NaCl	0.15	0.075	n.m.	0.40
	1.0	0.17	n.m.	n.m.
KCl	0.01	0.027	0.09	0.15
	0.05	0.090	0.21	0.37
	0.15	0.15	0.32	0.52
	0.5	0.20	0.50	1.0
	1.0	0.30	0.75	1.5
	3.0	0.80	1.8	3.9
RbCl	0.15	0.15	n.m.	0.55
CsCl	0.15	0.14	n.m.	0.57
KCH_3_COO	0.15	0.14	0.27	0.48
N(CH_3_)_4_Cl	0.15	0.040	n.m.	0.25*
N(C_2_H_5_)_4_Cl	0.15	0.020	n.m.	0.16*
Tris-HCl	0.15	0.020	n.m.	0.084

a The membranes were formed from 1% (mass/volume) asolectin dissolved in n-decane. The aqueous solutions were unbuffered and had a pH of 6. The applied voltage was 20 mV, and the temperature was 20°C. The average single-channel conductance, *G* (i.e. current divided by voltage), was calculated from at least 80 single events. The standard deviation of the single-channel conductance was generally below ±15%. *c* is the concentration of the aqueous salt solutions. The single-channel conductance of wildtype HlyA of *E. coli* is given for comparison [28]. The values denoted with an asterix were measured during this study with purified HlyA. n.m. means not measured.

### Selectivity of the HlyA_Δ71–110_ and HlyA_Δ264–286_ channels

Zero-current membrane potential measurements were performed to obtain further information on the molecular structure of the channels formed by HlyA_Δ71–110_ and HlyA_Δ264–286_. Asolectin membranes were formed in 50 mM salt solution and the mutant proteins were added to the aqueous phase when the membranes were in the black state. After incorporation of 100 to 1000 channels into a membrane, ten-fold salt gradients were established by addition of small amounts of concentrated salt solution to one side of the membrane. For all salts tested in these experiments (KCl, LiCl, and KCH_3_COO), the more diluted side of the membrane became positive, which indicated preferential movement of cations through the HlyA_Δ71–110_ and HlyA_Δ264–286_ channels, i.e. the channels are cation-selective as suggested from the single-channel data. The zero-current membrane potentials for ten-fold salt gradients were always around 40 mV. Their analysis using the Goldman-Hodgkin-Katz equation [50] suggested that anions could also have a certain permeability through the hemolysin channels because the permeability ratios *P*_cations_/*P*_anions_ were around 10. However, the permeability ratios appeared only little changed as compared to wildtype HlyA. Furthermore, the asymmetry potentials were very similar for the three salts composed of anions and cations of different mobility. This means probably that the HlyA_Δ71–110_ and the HlyA_Δ264–286_ channels are ideally selective for cations because of negative charges attached to the channel similar as has been found for wildtype HlyA [28] (see also Discussion).

### Osmotic protection experiments

In previous studies it has been questioned whether *E. coli* HlyA forms defined channels in lipid bilayers and erythrocyte membranes [36], [37]. We performed osmotic protection experiments to check whether HlyA, HlyA_Δ71–110_, and HlyA_Δ264–286_ form defined channels in sheep erythrocyte membranes. Of further interest was the size of the HlyA_Δ71–110_ and HlyA_Δ264–286_ channels because the lipid bilayer studies described above suggested a smaller size of these channels as compared to those formed by wildtype HlyA. For the osmotic protection assays, sheep erythrocytes either suspended in saline solution or in saline solution supplemented with 30 mM carbohydrates of different molecular masses were incubated with HlyA, HlyA_Δ71–110_, and HlyA_Δ264–286_. For short incubation times of about 20 min or for small HlyA concentrations (50 ng/ml), melezitose (diameter 1.14 nm) but neither arabinose (diameter 0.62 nm) nor cellobiose (diameter 0.92 nm) tended to protect the erythrocytes from osmotic lysis by HlyA, HlyA_Δ71–110_, and HlyA_Δ264–286_. However, at incubation times of 60 min and at hemolysin concentrations of 500 ng/ml none of these carbohydrates could protect the sheep erythrocytes from lysis. This means probably that all carbohydrates used in this and in a previous study [36] were able to pass the HlyA channel. Another possibility, however, was that the HlyA channel was indeed not defined in size as has been suggested previously [36] and that HlyA acted similar to a detergent to partially lyze the erythrocyte membrane.

**Figure 5:**
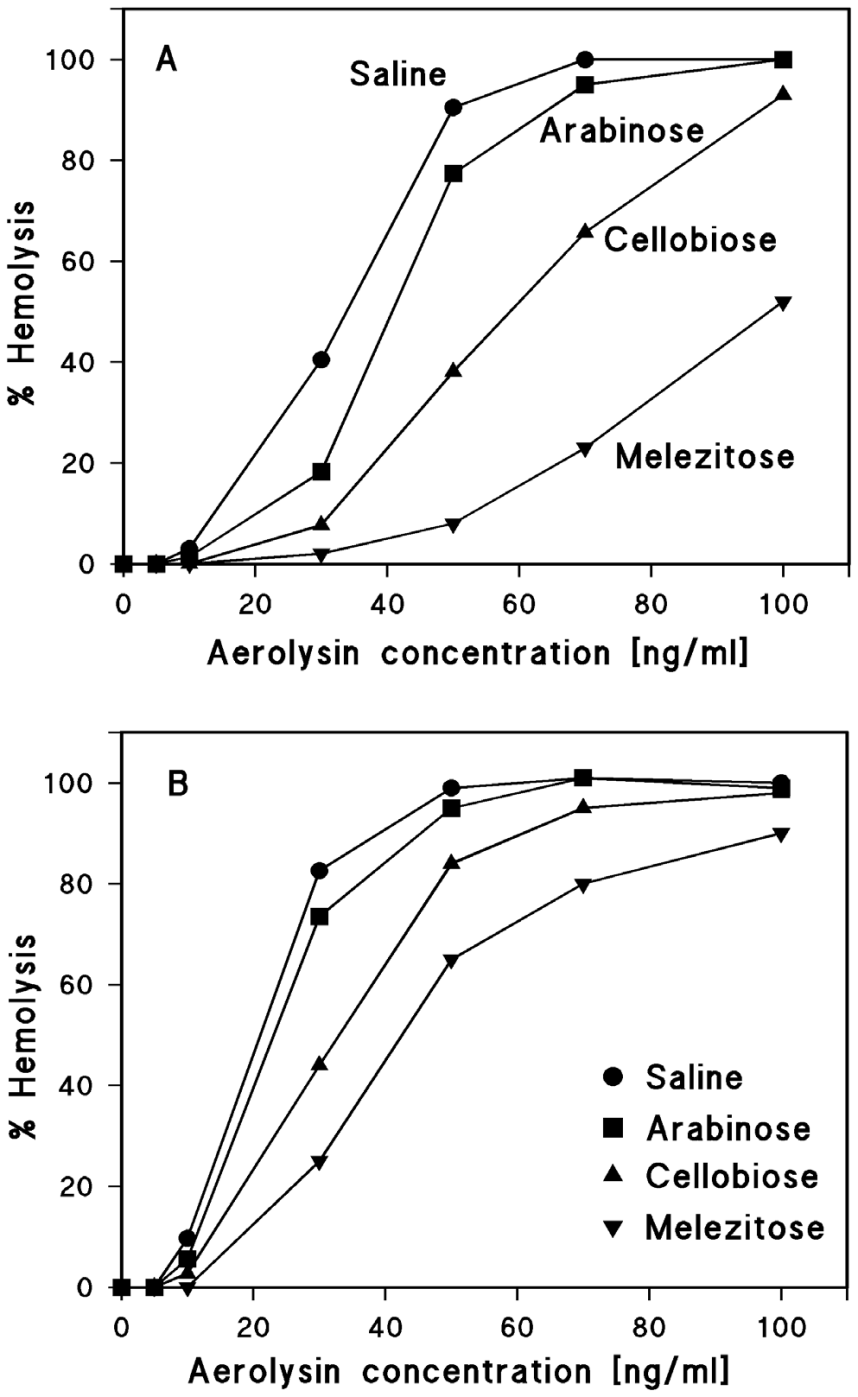
Results of osmotic protection experiments with aerolysin of *A. sobria*. Sheep erythrocytes in saline solution (control) or in saline solution supplemented with 30 mM of different carbohydrates (arabinose, cellobiose, and melezitose, with diameters of 0.62, 0.92, and 1.14 nm, respectively) were incubated with the toxin at 37°C for 30 min (A) and 90 min (B). Erythrocyte lysis was determined as a function of increasing aerolysin concentrations.

To check such a possibility, we performed osmotic protection experiments with another cytolytic toxin, aerolysin from *Aeromonas*, which forms stable heptamers in the presence of lipids and has a well-defined channel size [39], [55]–[58]. Osmotic protection experiments of the same type as described above for HlyA resulted in a partial protection of sheep erythrocytes towards aerolysin-mediated lysis for toxin concentrations up to 100 ng/ml when the saline solution was supplemented with 30 mM melezitose and when the cells were incubated with the toxin for 30 min at 37°C (Fig. 5A). However, incubation of the sheep erythrocytes for 90 min with aerolysin resulted in almost complete lysis in the presence of 30 mM melezitose (Fig. 5B). This result does not mean that the size of the aerolysin channels is time-dependent. It simply means that the permeability of the aerolysin channels for melezitose is not sufficient at a 30 min time scale to induce erythrocyte lysis, probably because the size of the aerolysin channel is close to the diameter of melezitose (1.14 nm).

**Figure 6:**
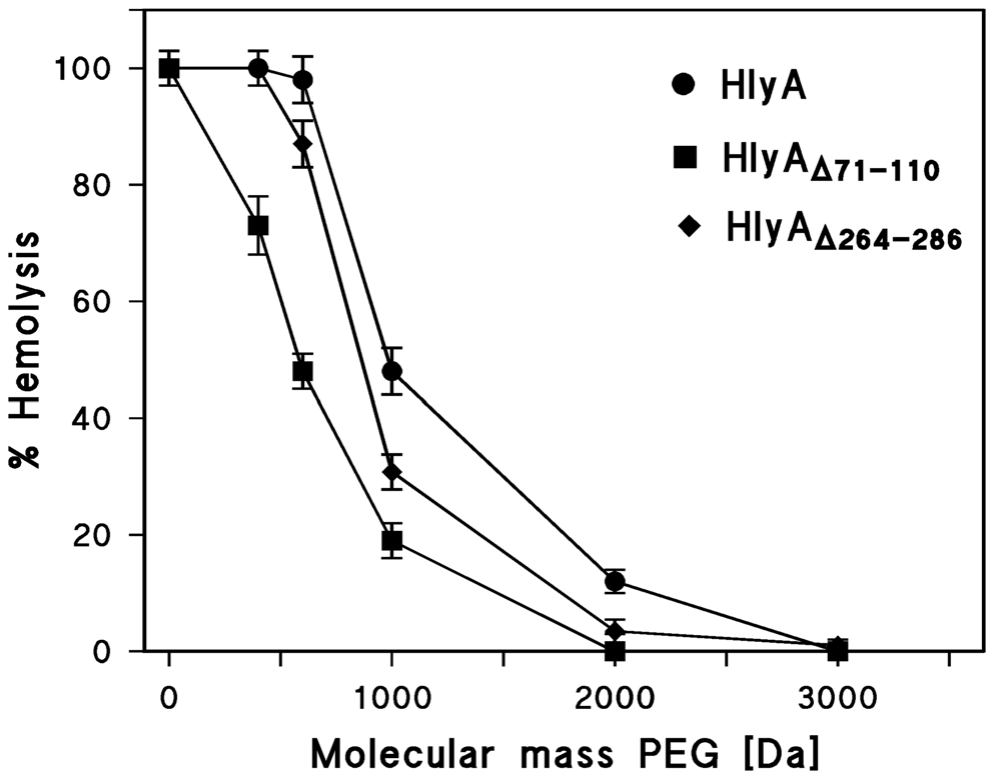
Results of osmotic protection experiments with HlyA, HlyA_Δ71–110_, and HlyA_Δ264–286_. Sheep erythrocytes were incubated with the toxins at 37°C for 60 min in saline solution (control) or in saline solution supplemented with 30 mM of PEGs of different molecular masses (PEG 400, 600, 1000, 2000, 3000, and 4000, with diameters of 1.07, 1.32, 1.72, 2.47, 3.05, and 3.54 nm, respectively). The concentration of HlyA was 0.5 µg/ml and that of HlyA_Δ71–110_ and HlyA_Δ264–286_ 2.5 µg/ml. The degree of hemolysis is shown as a function of the molecular mass of the PEGs.

Our data suggest that all carbohydrates used here are sufficiently small to pass through the defined channels formed by HlyA, HlyA_Δ71–110_, and HlyA_Δ264–286_. To elucidate the size of all three channels in more detail, we performed osmotic protection experiments with other solutes and supplemented the saline with 30 mM PEG of different molecular masses (400, 600, 1000, 2000, 3000, and 4000 Dalton, with diameters of 1.07, 1.32, 1.72, 2.47, 3.05, and 3.54 nm, respectively). To avoid any interference with hemolysin concentration and incubation time, we used in the case of wildtype HlyA the very high HlyA concentration of 0.5 µg/ml and incubated the cells for 60 min with the toxin at 37°C. Above that concentration and for longer assay times we did not observe much difference in the protection experiments, i.e. the degree of lysis was virtually constant and about 100% for small solutes. Fig. 6 shows the results of these experiments. The maximum erythrocyte lysis was plotted against the molecular mass of the PEGs. PEG 400 and PEG 600 did not protect the sheep erythrocytes from lysis by HlyA in agreement with the experiments using carbohydrates. PEG 1000 and PEG 2000 showed partial protection, whereas no lysis was observed for PEG 3000 and PEG 4000.

We performed similar measurements with HlyA_Δ71–110_ and HlyA_Δ264–286_ and included the data in Fig. 6. However, since the hemolytic activity of these HlyA mutants was much smaller than that of HlyA, we had to use a much higher concentration to reach full hemolysis in the different solutions. At a mutant HlyA concentration of 2.5 µg/ml and 60 min incubation time, virtually stable conditions were obtained for sheep erythrocyte lysis. As shown in Fig. 6, PEG 400, 600, and 1000 (diameters 1.07, 1.32, and 1.72 nm, respectively) achieved already partial protection in the case of HlyA_Δ71–110_. Starting with PEG 2000 (diameter 2.47 nm), no lysis of sheep erythrocytes by HlyA_Δ71–110_ was observed, which means that this solute cannot pass the mutant hemolysin channel. Similarly, the addition of PEG 600 and PEG 1000 resulted already in partial protection of the erythrocytes in the case of HlyA_Δ264–286_, but PEG 2000 did not provide full protection, indicating that the channel diameter of this HlyA mutant was slightly larger than that of HlyA_Δ71–110_. These results clearly indicate that the deletion of the amino acid residues 71–110 and 264–286 of HlyA decreased its channel size.

## Discussion

The results presented here confirm the notion that *E. coli* HlyA forms defined transmembrane channels with a diameter between 2 and 3 nm in both lipid bilayers and erythrocyte membranes. In addition, we present some evidence that β-strands could be involved in channel formation by HlyA. In bilayers formed from asolectin, the HlyA channel has an open state single-channel conductance of about 520 pS in 150 mM KCl [28], [59]. Several other RTX toxins (ApxI of *Actinobacillus pleuropneumoniae*, HlyA of *Proteus vulgaris* and *Morganella morganii*, EHEC-hemolysin of enterohemorrhagic *E. coli*) form channels of similar conductance [29], [60], [61]. The data clearly suggest that these toxins do not have a detergent-like activity as this would result in fuzzy and not well-defined channels. The osmotic protection experiments performed here and elsewhere [20] also suggest a defined channel formation for *E. coli* HlyA. Our experiments with *Aeromonas* aerolysin, a toxin known to form a fixed channel following heptamerization [39], [55]–[58], further supported this view by showing that osmotic protection depends on toxin concentration and incubation time even if a defined channel was generated.

In a study using pure phosphatidylcholine bilayers and different spectroscopic methods, no indication for a transmembrane arrangement of HlyA has been found [37]. Instead, it has been suggested that HlyA inserts only into the outer leaflet of the lipid bilayer, thereby causing destabilization and transient breakdown of the membrane. However, spectroscopic analyses of the type used previously [37] are probably not suited to identify and study the small membrane-spanning portion of a huge channel-forming complex, because most of the material may be localized on the membrane surface. As an example, in the case of α-hemolysin (α-toxin) from *Staphylococcus aureus*, which is not related to *E. coli* HlyA, the channel is formed by a heptamer of about 232 kDa, but less than 200 amino acid residues (14 β-strands each about 10 residues long, corresponding to about 15 kDa) are localized within the membrane [62], [63].

The structure of the *E. coli* HlyA pore is largely unknown although it seems to be formed by a toxin oligomer as also suggested for CyaA of *B. pertussis* [28], [30], [32], [33], [64]. An X-ray structure is so far unavailable for *E. coli* HlyA. Based on results of site-directed mutagenesis studies and secondary structure predictions, it has previously been suggested that the pore-forming domain of HlyA includes hydrophobic and/or amphipathic α-helices located in the hydrophobic region (residues 238–410) [14]–[16], [19]. Studies employing cysteine scanning mutagenesis and membrane insertion-dependent labeling further suggested that the hydrophobic region of HlyA is the principal region that inserts into the membrane [17]–[19]. An amphipathic helix involved in pore formation was recently proposed to exist particularly between the residues 272 and 298 [19], which was corroborated by the finding that a double substitution at positions 284 and 287 by proline, a known helix breaker [65], [66], abolished the lytic activity of HlyA without affecting binding to membranes [19]. It is remarkable in this context that a number of PFTs are thought to use α -helices for membrane insertion, but only for one of these α-PFTs, cytolysin A (ClyA) from *E. coli* (a toxin not related to *E. coli* HlyA), the crystal structure of the pore form is known [67]. Several other PFTs, on the other hand, such as α-hemolysin from *S. aureus* [62], [63], aerolysin from *Aeromonas* [56]–[58], or anthrax toxin protective antigen from *Bacillus anthracis* [68], [69] form transmembrane β-barrels composed of amphipathic β-strands and are designated as β-PFTs [70], [71].

To address the question whether the pore-forming domain of *E. coli* HlyA possibly contains β-strands in addition to α-helices, we searched within the first 300 residues of HlyA for amphipathic β-strands similar to those in bacterial outer membrane proteins using the two public domain programs PRED-TMBB and TMBETA-NET. The analyses led to the prediction of at maximum 13 such segments, some of which were, however, rather short, which means that they are hardly membrane spanning. According to the secondary structure prediction, we constructed four HlyA mutants exhibiting deletions of the residues 71–110, 158–167, 180–203, and 264–286. These regions contain either one or two (residues 71–110) possible amphipathic transmembrane β-strands. The deletion in HlyA_Δ264–286_ obviously overlaps with the presumed α-helix from residue 272 to 298 suggested by Valeva *et al.* [19], but our analyses predicted a β-strand spanning the residues 272 to 283. In addition, the contribution of the G284P substitution to the loss of lytic activity observed for HlyA_G284P/I287P_ [19] is unclear so far.

HlyA_Δ71–110_, HlyA_Δ158–167_, HlyA_Δ180–203_, and HlyA_Δ264–286_ were overproduced in *E. coli* 5K and were secreted from the *E. coli* cells in levels similar to wildtype HlyA, but they showed strongly reduced hemolytic activity, indicating that either binding to erythrocytes and/or pore formation was impaired or that the pore structure was altered. Lipid bilayer experiments using asolectin membranes revealed that two of these HlyA mutants, HlyA_Δ71–110_ and HlyA_Δ264-286_, formed cation-selective channels that resembled those formed by wildtype HlyA but had a much smaller conductance. This suggested that the deletions in these two mutants led to a smaller channel size without causing a substantial change of the HlyA structure, which probably would have resulted in the complete inhibition of channel formation or in gross disturbance of the channel structure as was observed for the other two mutants, HlyA_Δ158–167_ and HlyA_Δ180–203_. The data rather suggested that part of the channel-forming domain was missing in HlyA_Δ71–110_ and HlyA_Δ264–286_, possibly one or two β-strands. It is noteworthy that we obtained virtually the same results in lipid bilayer experiments using culture supernatants, precipitated proteins or purified toxins, which makes artifacts such as the aggregation of HlyA and its mutants rather unlikely. The lipid bilayer studies also demonstrated that HlyA_Δ71–110_ and HlyA_Δ264–286_ formed approximately the same number of channels as HlyA when the same protein concentration was used, indicating that their channel-forming activity was not significantly affected. Taken together, the data thus suggested that the very weak hemolytic activity of these two mutants is presumably caused by the smaller size of the mutant channels. It is remarkable that other RTX toxins showing a small single-channel conductance (about 100 pS or less in 150 mM KCl), such as CyaA of *B. pertussis* [30], [31], ApxIII of *A. pleuropneumoniae* [60], or leukotoxin of *Mannheimia haemolytica* (formerly *Pasteurella haemolytica*) (Maier and Benz, unpublished data), have a similarly low hemolytic activity as found here for HlyA_Δ71–110_ and HlyA_Δ264–286_, or are nonhemolytic [72].

Osmotic protection experiments suggested that the channels formed by HlyA, HlyA_Δ71–110_, and HlyA_Δ264–286_ were large enough to allow passage of all carbohydrates used in this study. Similar experiments with PEGs of different molecular masses, on the other hand, allowed to roughly iestimating the channel size for HlyA and the two HlyA mutants. PEG 3000 (diameter about 3.05 nm) provided full protection against hemolysis by wildtype HlyA, which means that the HlyA channel diameter is probably smaller than 3 nm. PEG 2000 (diameter 2.47 nm) completely blocked the hemolysis by HlyA_Δ71–110_ which still showed a certain permeability for PEG 1000 (diameter 1.72 nm), suggesting a channel diameter of about 2 nm for this mutant. The HlyA_Δ264–286_ channel appeared to be somewhat larger than that of HlyA_Δ71–110_ because PEG 2000 showed some minor permeability through the channel. These results argue indeed that removal of the amino acid residues 71–110 and 264–286 from HlyA decreased the size of the hemolysin channel.

**Figure 7:**
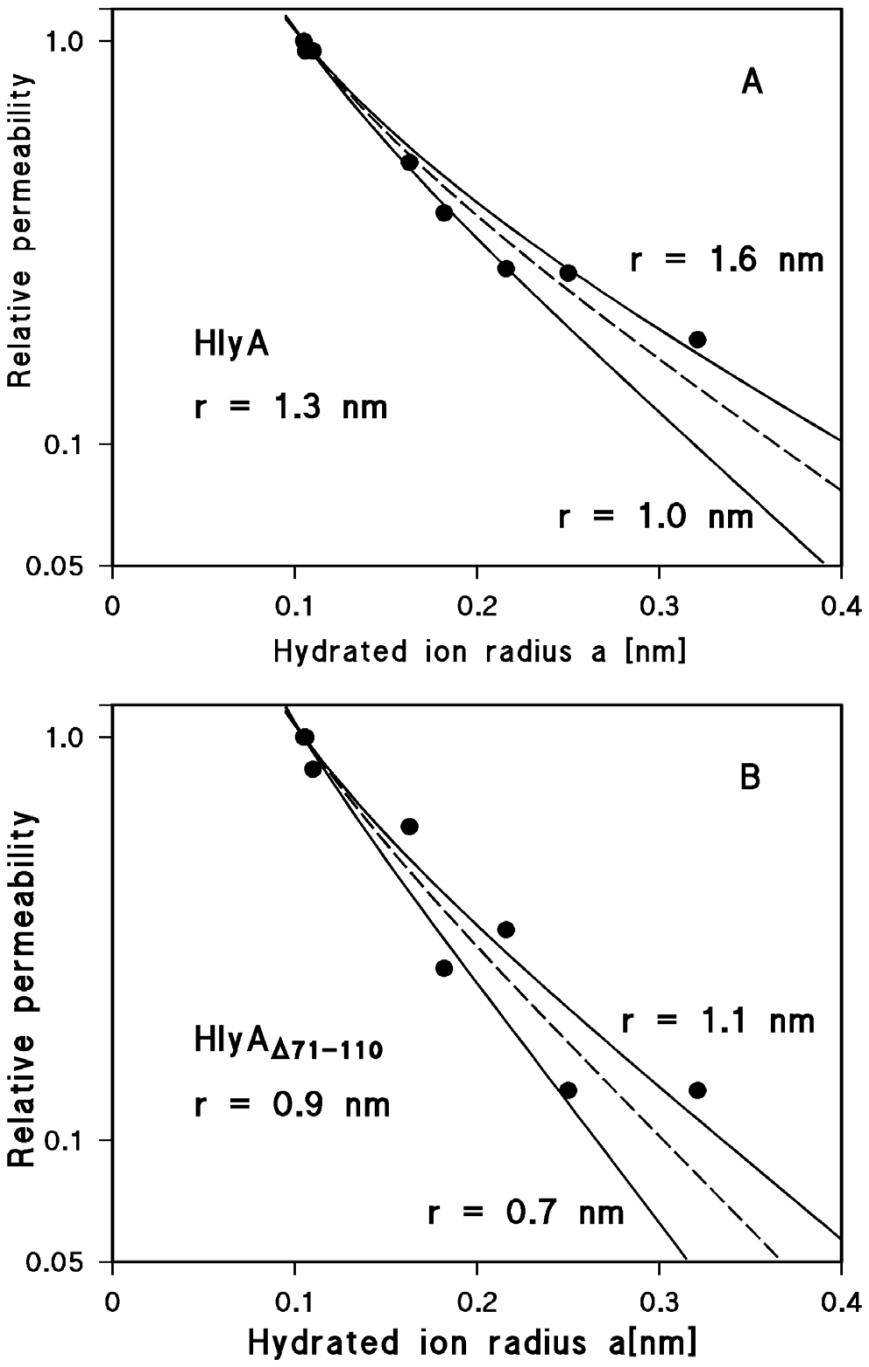
Calculation of the channel diameters of HlyA and HlyA_Δ71–110_ from the single-channel conductance. The single-channel conductance data of HlyA and HlyA_Δ71–110_ were fitted by using the Renkin correction factor multiplied by the aqueous diffusion coefficients of the different cations. The single-channel conductance for the different cations taken from Table 2 was normalized to that observed for Rb^+^ (hydrated ion radius 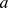 = 0.105 nm), which was set to 1.0, and plotted versus the hydrated ion radii taken from Table 3 of Maier *et al.* [60]. The points correspond to the single-channel conductance observed with Li^+^, Na^+^, K^+^, Cs^+^, N(CH_3_)_4_^+^, N(C_2_H_5_)_4_^+^, and Tris^+^, which were all used for the pore diameter estimation (see Discussion). (A) The fit (solid lines) is shown for wildtype HlyA channels with *r* = 1.6 nm (upper line) and *r* = 1.0 nm (lower line). The best fit was achieved with *r* = 1.3 nm (diameter = 2.6 nm), which corresponds to the broken line. (B) The fit (solid lines) is shown for the HlyA_Δ71–110_ channels with *r* = 1.1 nm (upper line) and *r* = 0.7 nm (lower line). The best fit of all data was achieved with *r* = 0.9 nm (diameter = 1.8 nm), which corresponds to the broken line.

It was also possible to calculate the channel diameters of HlyA and HlyA_Δ71–110_ from their single-channel conductance (see Materials and Methods). Since the channels formed by both proteins are strongly cation-selective, the conductance data were used to estimate the relative permeability of the different cations through the channels. The single-channel conductance for the different cations taken from Table 2 were for this purpose normalized to that for Rb^+^ and plotted as a function of the hydrated ion radii (Fig. 7). Fig. 7A shows the fit of the relative cation permeability through wildtype HlyA channels (as calculated from the single-channel data) with the aqueous diffusion coefficients, *D*, of the ions multiplied by the Renkin correction factor (eqn. (1); see Materials and Methods). The best fit was obtained with *r* = 1.3 nm, corresponding to a channel diameter of 2.6 nm (the data lie between the channel radii *r* = 1.0 nm and *r* = 1.6 nm). It is noteworthy that such a channel diameter agrees very well with the diameter derived from the osmotic protection experiments described here and elsewhere [20]. In the case of HlyA_Δ71–110_, the best fit of the relative cation permeability with *D* multiplied by the Renkin correction factor was obtained using *r* = 0.9 nm, corresponding to a channel diameter of 1.8 nm (the data lie between *r* = 0.7 nm and *r* = 1.1 nm) (Fig. 7B). This suggests that the channel formed by HlyA_Δ71–110_ is indeed considerably smaller than that of wildtype HlyA. The single-channel data thus show satisfactory agreement with the smaller hemolytic activity of this mutant and the results of the osmotic protection experiments.

**Figure 8:**
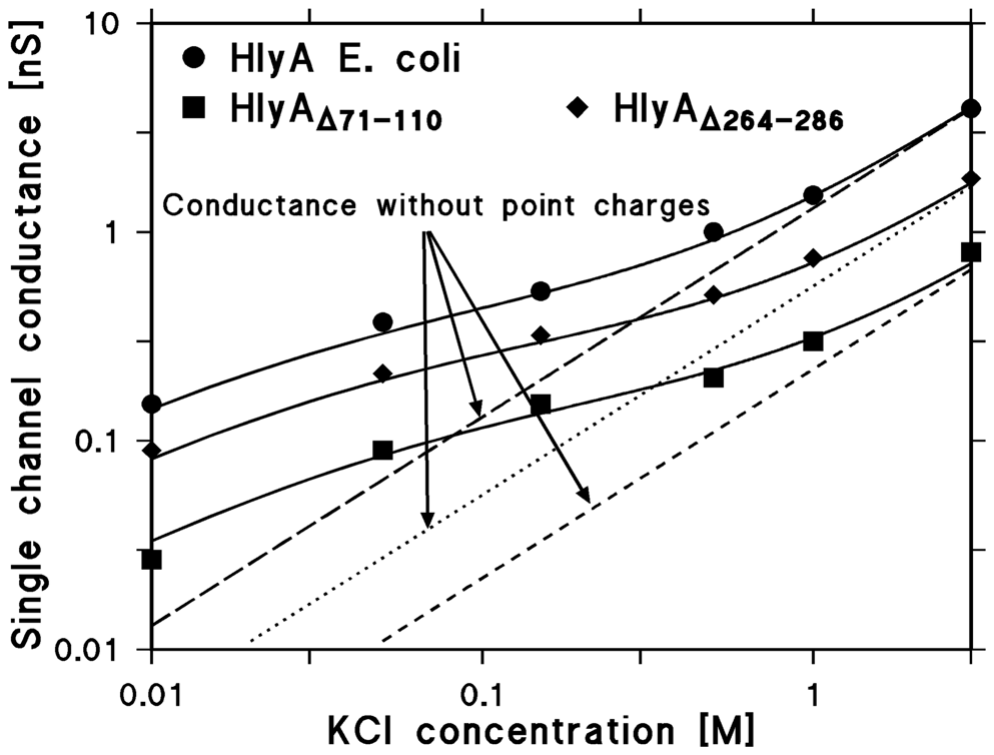
Effect of charges in wildtype and mutant HlyA on the single-channel conductance. The single-channel conductance of the HlyA, HlyA_Δ71–110_, and HlyA_Δ264–286_ channels is shown as a function of the KCl concentration in the aqueous phase. The solid lines represent the fit of the single-channel conductance data with eqn. *G(c) = G_0_⋅c_0_^+^* (a combination of eqs. (3–5 and 7) assuming the presence of negative point charges within the channel (for HlyA: 2.3 negative charges, *q = *−3.68×10^–19^ As; for HlyA_Δ71–110_∶1.7 negative charges, *q* = -2.72×10^–19^ As; for HlyA_Δ264–286_∶2 negative charges, *q = *-3.2×10^–19^ As) and assuming a channel diameter of 2 nm, 1.4 nm, and 1.6 nm for HlyA, HlyA_Δ71–110_, and HlyA_Δ264–286_, respectively. *c*, concentration of the KCl solution in M (molar); *G*, average single-channel conductance in nS (nano Siemens, 10^–9^ S); *G_0_*, specific single-channel conductance in the absence of negative point charges given in pS/M. The broken, dotted, and fractured (straight) lines show the single-channel conductance of the HlyA, HlyA_Δ264–286_, and HlyA_Δ71–110_ channels in the absence of point charges and correspond to linear functions between channel conductance and bulk aqueous concentration (eqn. (7); *G(c) = G_0_⋅c*).

The data shown in Table 2 demonstrate that the single-channel conductance of HlyA, HlyA_Δ71–110_, and HlyA_Δ264–286_ are not linear functions of the bulk aqueous salt concentration. Instead, a slope of about 0.5 to 0.6 was observed on a double-logarithmic scale for the conductance versus concentration curves (Fig. 8). This indicated that charge effects caused by negatively charged groups influence the properties of these proteins. The charges result in a substantial ionic strength-dependent potential inside the channels, which attracts cations and repels anions. Accordingly, it influences both single-channel conductance and zero-current membrane potential. In particular, its conductance is at low ionic strength larger than expected from the channel dimension. The Debeye-Hückel theory provides a quantitative description of the effect of charges on counterions in an aqueous environment. A description of the effect of point charges on a membrane surface on counterion accumulation is given by the treatment of Nelson and McQuarrie [73], which in principle does not consider charges attached to a channel. However, we assume here that the negatively charged groups are localized at the toxin channel.

In case of a negative point charge, *q*, in an aqueous environment a potential *φ* is caused that is dependent on the distance, *r*, from the point charge:
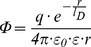
(3)


ε_0_ ( = 8.85×10^–12^ F/m) and ε ( = 80) are the absolute dielectric constant of vacuum and the relative dielectric constant of water, respectively, and *l_D_* is the Debeye length that controls the decay of the potential (and of the accumulated positively charged ions) in the aqueous phase:
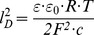
(4)where *c* is the bulk aqueous salt concentration, and *R*, *T*, and *F* (*RT/F* = 25.2 mV at 20°C) have the usual meaning. The potential *φ* created by a negative point charge on the surface of a membrane is twice that of eqn. (3) due to the generation of an image force on the opposite site of the membrane. The concentration of cations near the point charge, *c_0_^+^*, increases because of the negative potential. *c_0_^+^* is in both cases (Debeye-Hückel or Nelson-McQuarrie) dependent on the potential *φ* and given by:



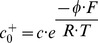
(5)Similarly, the anion concentration near the point charge, *c_0_* ^–^, decreases according to:
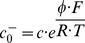
(6)


In the following, we assume that the negative point charge is attached to the channel. In such a case, the channel conductance is limited by the accumulated positively charged ions and not by their bulk aqueous concentration. The single-channel conductance, *G*, as a function of the ion concentration is given by the linear function:
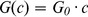
(7)where *G_0_* is the specific single-channel conductance (i.e. the slope of the conductance-concentration curve). Eqn. (5) can be introduced into eqn. (7), and we can try to fit the non-linear concentration dependence of the single-channel conductance of HlyA, HlyA_Δ71–110_, and HlyA_Δ264–286_ given in Table 2 with eqn. *G(c) = G_0_⋅c_0_^+^*, using the eqs. (3) to (5) and (7) and the Nelson-McQuarrie formalism [73]. In Fig. 8, the fit of the conductance data of the three proteins with eqn. *G(c) = G_0_⋅c_0_^+^* is shown by solid lines. In the case of HlyA, using *G_0_* = 1.3 nS/M, a best fit was obtained by assuming that 2.3 negatively charged groups (*q = -3.68×10^–19^ As*) are located at the pore mouth and that the channel radius is about 1 nm. For HlyA_Δ71–110_, using *G_0_* = 0.22 nS/M, a best fit was achieved with 1.7 negatively charged groups (*q = -2.72×10^–19^ As*) and a channel radius of about 0.7 nm, again suggesting that the HlyA_Δ71–110_ channel is much smaller than that of HlyA. The smaller number of charges agrees well with the removal of three net negatively charged groups by the deletion of residues 71–110. In the case of HlyA_Δ264–286_, using *G_0_* = 0.55 nS/M, a best fit of the conductance data was achieved assuming 2 negatively charged groups (*q = −3.2×10^–19^ As*) and assuming a channel radius of about 0.8 nm, again clearly smaller as for HlyA. Fig. 8 also shows the single-channel conductance calculated for the three proteins in the absence of point net charges, corresponding to eqn. (7), i.e. assuming the same *G_0_* values as mentioned above, but with *c* given by the bulk aqueous concentration (see the broken, dotted, and fractured lines in Fig. 8). A comparison with the solid lines indicates that in all three cases the charges attached to the channel substantially influence the conductance at lower bulk aqueous concentrations, while their influence is rather small at high ionic strength. The number of negative charges involved in the accumulation of cations at the channel mouth is not very precise, because the dielectric constant of their environment is not known. When the dielectric constant is low, then the Nelson-McQuarrie formalism [73] has to be applied and *q* in eqn. (3) has to be replaced by 2⋅*q.* In the case of high dielectric environment, the Debeye-Hückel theory is valid. The estimated channel radius is more precise as has also been demonstrated elsewhere [53]. It is noteworthy that the effect of charges near the channel mouth has also been theoretically predicted and experimentally verified in other investigations [74]–[76] and indicate that strategically placed charges near a channel can lower its energy barriers and accumulate ions to guide them through the channel.

In conclusion, the finding that two HlyA mutants with deletions of small regions predicted to form amphipathic β-strands generated only very small (but apart from that largely normal) channels suggests that these deletions removed part of the channel-forming domain and that β -strands may play a role in channel formation by HlyA. However, since the formation of β-strands in the deleted regions has not been shown experimentally, we can at present only speculate that the HlyA channel might contain amphipathic β-strands. Furthermore, given that putatively α-helical structures in the hydrophobic region of HlyA have been found to be important for pore formation [15], [16], [19], it appears unlikely that the channel is formed exclusively from β-strands. It is rather tempting to hypothesize that *E. coli* HlyA might use a combination of α-helices and β-strands for channel formation. Further investigations are required to verify such a possibility.
